# Effect of Culture Media on the Yield and Protein Content of *Pleurotus ostreatus* (Jacq.) Kumm Mycelia

**DOI:** 10.1155/ijfo/5562732

**Published:** 2024-12-27

**Authors:** Kayise Hypercia Maseko, Thierry Regnier, Obiro Cuthbert Wokadala, Paul Bartels, Belinda Meiring

**Affiliations:** ^1^Department of Biotechnology and Food Technology, Tshwane University of Technology, Pretoria, South Africa; ^2^Faculty of Agriculture and Natural Sciences, University of Mpumalanga, Nelspruit, South Africa; ^3^WildBio Company, Pretoria, South Africa

**Keywords:** media optimization, mycelium cultivation, oyster mushroom, protein

## Abstract

The development of alternative proteins derived from fungi-based sources is gaining recognition due to their health benefits and lower environmental impact, compared to traditional animal-based sources. In this study, we investigated the culture conditions for *Pleurotus ostreatus* mycelia, focusing on the nutritional requirements and yield optimization using solid surface culture and liquid-state culture methods. Our findings indicate that optimal culture conditions involve glucose as the primary carbon source, with an initial pH of 6.0. By the eighth day of the culture period, media formulated with amaranth seed flour and Bambara groundnut flour yielded the highest mycelial protein content, characterized by a compact filamentous network with fewer open pores. Additionally, urea supplementation at 0.01% concentration on amaranth seed–based nutrient medium significantly increased the protein content from 31.4% to 38.7% (dry weight basis). The research findings contribute to the development of fungal-based proteins, which are essential in the production of sustainable food products.

## 1. Introduction

Due to the increase in demand and market value of alternative proteins, the cultivation of fungi-based ingredients has become an appealing option [1]. In addition to its health and nutraceutical qualities, the production of fungi plays a role in achieving environmental sustainability [2]. As an alternative to macrofungi, efficient production of edible mushroom mycelia has been considered important and has gained interest in controlling and improving culturing technology over the years [3].

Mycelia can be developed as a dietary supplement that provides nutraceutical characteristics to food products. They can also act as a nutrition supplement for malnourished individuals or as an alternative protein source. To resolve upscaling challenges, mycelia must be produced and available all year while being economically viable despite the cost of culture media [2]. Upscaling the production of mycelium biomass is required to provide a sustainable ingredient for the food industry [4].

Mushroom strains can grow on a variety of carbon, nutrient, and nitrogen sources [5]. In comparison to other edible fungi species, *Pleurotus* spp. require a short growth period. They can be cultivated using simple and inexpensive methods, with high adaptability, yield, and broader culture media utilization over a wide temperature range [6, 7]. *Pleurotus ostreatus*, commonly known as oyster mushroom, is one of the most important mushrooms in the global food sector. It is known for its distinct flavor and as a source of vitamins, minerals, protein, amino acids, and dietary fiber. In addition, *P. ostreatus* mycelium biomass has received particular interest due to its numerous applications in the food industry [8].

Due to the effect of culture media on mycelial growth rate and biomass yield, the media composition, including the pH, carbon, nutrient, and nitrogen sources, must be optimized in solid and liquid cultures [6]. Thus, the current study is aimed at improving the *Pleurotus* cultivation technology, by optimizing the culture conditions for mycelial yield and protein content.

## 2. Materials and Methods

### 2.1. Materials

The various materials used to prepare culture medium included amaranth seeds (*Amaranthus*), ragi or finger millet (*Eleusine coracana*) flour, jowar or sorghum (*Sorghum bicolor*) flour, and potato (*Solanum tuberosum*) flour which were purchased from a local supplier, the Wellness warehouse (Pretoria, South Africa). Digestive wheat bran (*Triticum aestivum*), flax seed (*Linum usitatissimum*) flour, and hemp seed (*Cannabis sativa*) powder were purchased from Dis-chem (Pretoria, South Africa). Bambara groundnuts (*Vigna subterranean*) were purchased from a local grocery store (Republic Fisheries and Meat Market, Pretoria, South Africa), whereas marula (*Sclerocarya birrea*) seed cake (a by-product after defatting the seeds) was obtained from Marula company (Phalaborwa, South Africa).

Glucose, fructose, maltose, sorbose, sucrose, casein hydrolysate, peptone powder, yeast extract powder, agar powder, and malt extract agar (MEA) were purchased from Sigma-Aldrich (Johannesburg, South Africa). All the other chemicals were of analytical grade and were purchased either from Sigma-Aldrich or Rochelle Chemicals (Johannesburg, South Africa).

### 2.2. Strain Inoculum Preparation

The edible mushroom fungal culture of *P. ostreatus* (HK35) obtained from Sylvan Incorporated (Centurion, South Africa) was used in this study. The pure strain culture was maintained and preserved on MEA in Petri dishes (covered with parafilm) in the dark at 4°C for 4 weeks and subsequently subcultured according to the method by Leong et al. [9], with modifications. Mycelial agar discs (6 mm in diameter) were obtained by puncturing the mycelia colonized plates (active physiological state) with a sterile glass Pasteur pipette in sterile conditions and used as inoculum in subsequent studies.

### 2.3. Evaluation of Optimal Growth Conditions for *P. ostreatus* Mycelium Using Solid Surface Culture

The optimal nutritional requirements and culture conditions for the growth of *P*. *ostreatus* mycelia were determined using the agar plate technique by the one-factor-at-a-time (OFAT) method [2, 10]. This approach includes systematically varying individual parameters while keeping others constant to assess their impact on the growth of mycelia. Thereafter, the experimental parameter level showing the highest growth diameter of the mycelia was selected as optimum. For each experimental analysis, a single *P*. *ostreatus* mycelial disc was inoculated on the center of Petri dishes (90 mm in diameter) containing the solidified medium (20 mL), sealed with parafilm, and incubated in a dark incubator at 25 ± 1°C. The diameter of the mycelium growth was measured every 2 days for 6 days. The mycelial growth characteristics (colony diameter and/or morphology) were observed for each analysis. The highest colony diameter was a direct measure of the suitability of each respective analysis.

The screening of five carbon sources at four concentrations was prepared according to the method by Hoa and Wang [5], with modifications. Basal medium (7 g potato flour, 1 g agar powder, and 0.50 g peptone powder, in 100 mL distilled water) was supplemented with each carbon source (glucose, fructose, maltose, sorbose, and sucrose), at various concentrations (2%, 4%, 6%, and 8%, *w*/*v*) before sterilization. A basal medium without carbon supplementation was used as a control.

The optimal pH requirement was determined (VioLab benchtop pH meter) by the effect of the five different initial pH levels on the growth rate of *P. ostreatus* [11]. Basal medium (7 g potato flour, 1 g agar powder, 0.50 g peptone powder, and 2 g glucose, in 100 mL distilled water) was prepared, and the pH of each medium was adjusted to pH 4.0, 5.0, 6.0, 7.0, and 8.0 with 1 M hydrochloric acid (HCL) or 1 M sodium hydroxide (NaOH) solution before sterilization.

Screening of nine nitrogen sources (ammonium chloride (NH_4_Cl), ammonium dihydrogen phosphate (NH_4_H_2_PO_4_), casein, malt extract, peptone, sodium nitrite, urea, wheat bran, and yeast extract) and two nutrient sources (ascorbic acid and calcium carbonate) was conducted at three concentration levels (0.01%, 0.05%, and 0.10%, *w*/*v* for micronutrients and 0.10%, 0.50%, and 1%, *w*/*v* for macronutrients) [5, 12]. Each source was supplemented on a basal medium (7 g potato flour, 1 g agar powder, and 2 g glucose, in 100 mL distilled water) before sterilization.

### 2.4. Evaluation of Growth Media for *P*. *ostreatus* Mycelium Using Liquid-State Culture

Mycelium production on 50 mL culture media was carried out in 250 mL Erlenmeyer flasks. The flask neck was plugged with cotton wool, covered with foil, and sterilized at 121°C for 15 min. Once sterilized, it was cooled down under a sterile flow of laminar air and inoculated with 10% (or 4 units of cutter round (6 mm)) mycelial disc inoculum. It was maintained as static culture in a dark incubator at 25°C ± 1°C. All cultivation experiments were conducted simultaneously in triplicate under the same conditions.

#### 2.4.1. Cultivation on Media With Various Nutrient Sources

Culture media were prepared in Erlenmeyer flasks from seven nutrient sources: amaranth seed (presoaked to reduce antinutritional components) flour, Bambara groundnut (presoaked to remove hull) flour, ragi flour, flaxseed flour, hemp seed powder, marula seed cake flour, and jowar flour. Media composed of 4 g of each nutrient source, 2 g glucose, and adjusted volume to 50 mL with distilled water. Media without glucose supplementation was used as a control. The media was sterilized, and *P. ostreatus* mycelial colonized discs were aseptically inoculated onto the media and incubated for up to 14 days.

#### 2.4.2. Cultivation on Nutrient-Based Media Supplemented With Various Concentrations of Nitrogen and Nutrient Sources

Media with supplement formulation was according to Bellettini et al. [6] with slight modifications. Media composed of 4 g of each nutrient source (amaranth seed flour and Bambara groundnut flour), additional supplementary nitrogen or nutrient source (measurements according to Table S1), and adjusted volume to 50 mL with distilled water in Erlenmeyer flasks. *P. ostreatus*-colonized mycelial discs were aseptically inoculated onto the media after sterilization and incubated for 8 days.

### 2.5. Assessment of Cultivation Parameters

The surface diameter of the mycelia colony growth on Petri dishes (90 mm) was measured using a digital caliper (millimeters) in the cross direction (horizontal and vertical measurements; three values were averaged) every 2 days for 6 days.

To monitor the rate of biomass growth, culture flasks were sacrificed at different time points (8, 10, 12, and 14 days) to harvest mycelium. The freshly harvested mycelia from the Erlenmeyer flasks were rinsed with sterile distilled water to remove medium components, excess liquid drained off, and biomass weighed. Mycelial biomass was frozen at −80°C for 24 h and lyophilized (−50°C for 24 h) in a Telstar (LyoQuest) laboratory freeze dryer to constant weight for dry-weight quantification (yield%). The yield (Equation (1)) was adapted from Debbarma, Sharma, and Kalia [13] and calculated to indicate how much the mycelium had grown relative to the growth media. This is from the average total weight of lyophilized mycelia per initial dry weight of growth media, employed in percentage as

(1) $$\begin{array}{c} \text{yield }\left(\%\right)=\frac{\text{dry mycelia weight }\left(\text{g}\right)}{\text{dry initial growth media weight }\left(\text{g}\right)}\times 100\% \end{array}$$

The protein yield generated (Equation (2)) was calculated based on the mycelial yield (grams) multiplied by the protein content (grams). The value obtained was divided by the incubation period (day) to determine the protein yield produced per day (Equation (3)).

(2) $$\begin{array}{c} \text{Protein yield }\left(\text{g}\right)=\text{yield }\left(\text{g}\right)\times \text{protein content }\left(\text{g}\right) \end{array}$$

(3) $$\begin{array}{c} \text{Protein yield per day }\left(\text{g}/\text{day}\right)=\frac{\text{protein yield }\left(\text{g}\right)}{\text{time period }\left(\text{days}\right)} \end{array}$$

### 2.6. Mycelium Analysis

#### 2.6.1. Morphology

To evaluate the structure of lyophilized mycelia [14], samples were mounted on aluminum stubs, exposing the top surface, and coated with carbon using a Quorum Q150T coating unit (Quorum, United Kingdom). The samples were visualized using a ZEISS Gemini 540 Ultra plus (Zeiss, Germany) field emission gun scanning electron microscope (FEG-SEM) at the Laboratory for Microscopy and Microanalysis, University of Pretoria (Pretoria, South Africa).

#### 2.6.2. Proximate Composition

The crude protein content of lyophilized mycelia samples was determined according to Association of Official Analytical Collaboration (AOAC) International–960.52 Dumas combustion method using Leco TruMac N (Michigan, United States). A protein conversion factor of 5.70 was used for mycelium samples.

### 2.7. Statistical Analysis

The optimal growth parameter data analysis was carried out using statistical analysis programs, in triplicate. The average results were reported and statistically significant differences by mean separation were performed using Duncan's multiple tests (*p* < 0.05) followed by post hoc tests. Analysis was performed using the Statistical Package for Social Sciences (SPSS) Program 29.0 software (International Business Machines Corporation (IBM) SPSS Inc., Chicago, United States). One-way analysis of variance (ANOVA) assessed the impact of variables, while partial least squares regression (PLS-R) analysis centered and weighted the two analytical values (control medium and medium with glucose) on the protein yield and responses.

## 3. Results and Discussion

### 3.1. Solid Surface Culture of *P*. *ostreatus*

#### 3.1.1. Effect of Various Carbon Sources and Concentrations on *P*. *ostreatus* Mycelial Growth Rate and Morphology

The five carbon sources affected *P. ostreatus* growth to a certain level when compared to the control medium, on the 6^th^ day of the culture period (active growth period). Akromah et al. [4] stated that carbon is the most important macronutrient for fungi since it promotes mycelial growth and increases mycelium network density. The results in Table 1 indicated that sucrose at 4% and 6% concentrations promoted the highest mycelial growth diameter (around 61 mm).

Maltose at 6% concentration promoted the growth diameter until 57.33 mm. The third highest mycelial growth diameter was in the case of media containing glucose (2%), sucrose (2%), and fructose (4%), reaching 57.33, 55.73, and 54.83 mm, respectively. The results of the highest mycelial growth could be attributed to the metabolite processes that support the carbon sources resulting in cellular energy that promotes the development of mass-branching hyphae [15]. However, when the sorbose concentration exceeded 6%, mycelial growth was inhibited substantially, when compared to the control medium.

**Figure 1:**
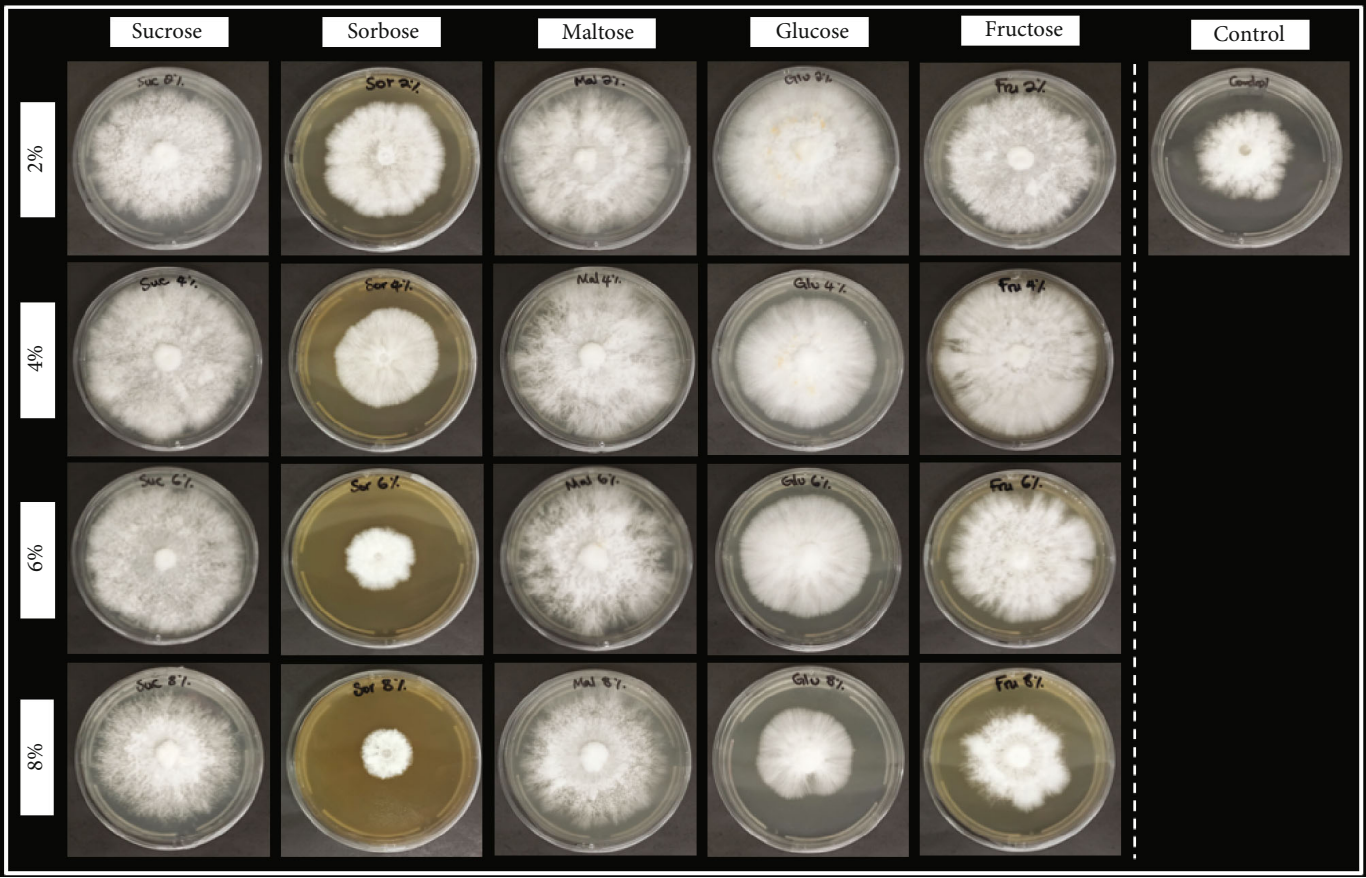
The appearance of *P*. *ostreatus* mycelial growth morphology impacted by the various carbon sources and concentrations at 8 days after inoculation.

Sucrose and glucose are the least expensive of the suitable carbon sources (glucose, maltose, and sucrose) that considerably support *P*. *ostreatus* growth. Glucose is required at a lower concentration than sucrose for optimal growth, while the mycelial morphology (Figure 1) for media supplemented with glucose as a carbon source shows a more compactly developed mycelium network. Therefore, glucose is observed as the most suitable carbon source for the culture of *P*. *ostreatus* mycelium, which is in accordance with the previous study by Bakratsas et al. [16]. Thus, glucose at 2% concentration was chosen as a carbon source in the subsequent experiments.

#### 3.1.2. Effect of Various pH Levels on *P*. *ostreatus* Mycelial Growth Rate and Morphology

Mycelial growth occurred at all pH levels as shown in Table 2, indicating the fungus' adaptability [4]; nonetheless, the optimal culture media pH condition is crucial for achieving optimum fungal growth and development [17]. On the 4^th^ day of the culture period, there was no significant difference (*p* > 0.05) among pH levels 5.0 and 7.0 which impacted the second highest mycelial growth rate (32.67–33.23 mm), and the pH levels 4.0 and 8.0 attributed to the lowest mycelial growth rate (29.33–30.80 mm). During the 6*^th^* day of the culture period, there was no significant difference in mycelial growth rate for pH 4.0, 5.0, and 7.0 levels; however, mycelial growth was the lowest at pH 8.0. This can be attributed to the findings that *Pleurotus* spp. mycelia grow quickly on a slightly acidic than basic medium [11].

**Figure 2:**
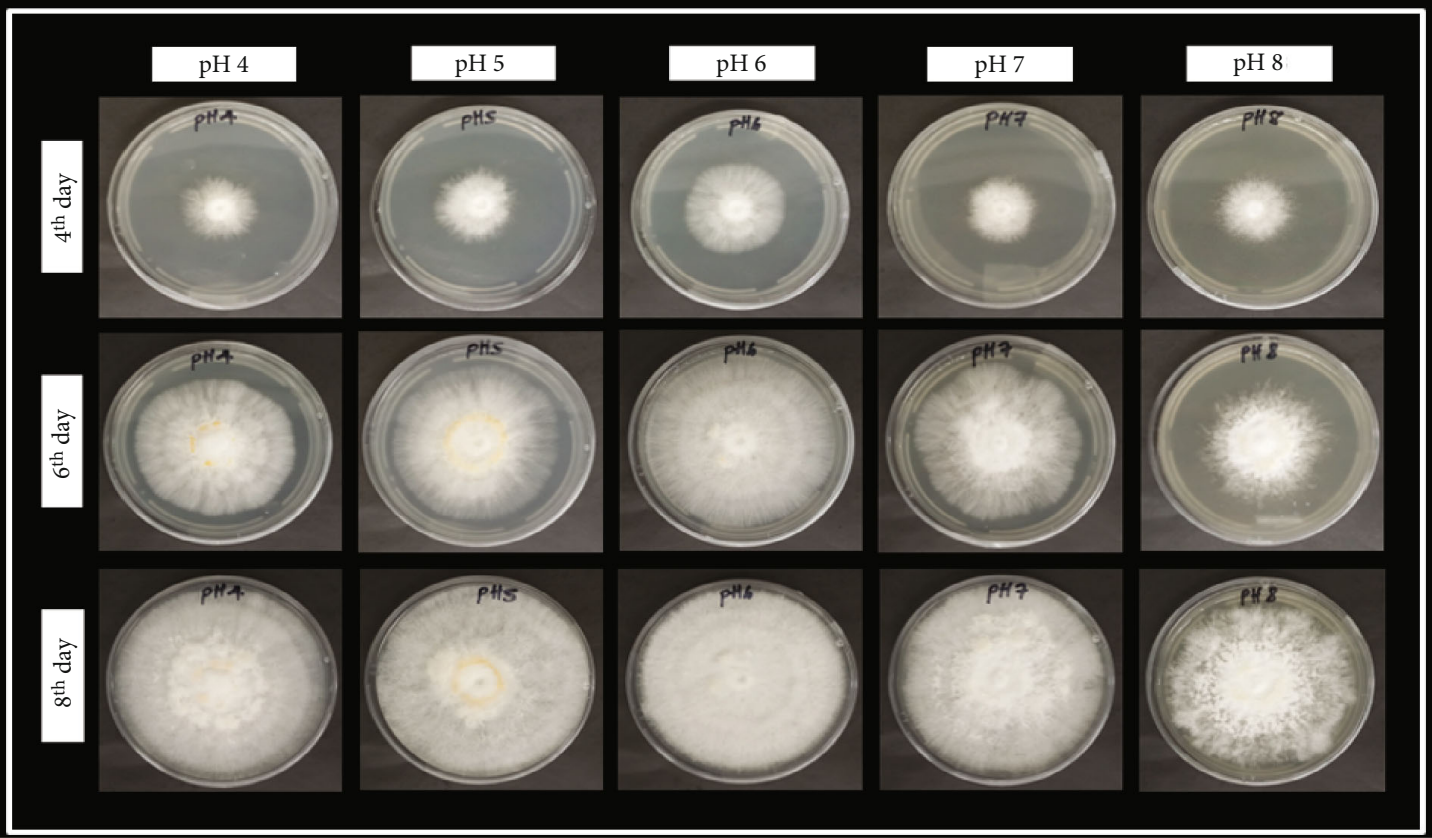
The appearance of *P*. *ostreatus* mycelial growth morphology affected by the various pH levels on basal media at 8 days after inoculation.

The highest mycelial growth rate of *P*. *ostreatus* was recorded at pH 6.0 on the 4^th^ and 6^th^ days of the culture period. As a result, pH 6.0 was identified to be the most favorable condition for optimal mycelial growth. These results are in accordance with a previous study on *P*. *ostreatus* by Hoa, C. Wang, and C. Wang [18], where the mycelial growth was maintained and optimum around pH 6.0. On the 8^th^ day of the culture period of this study, Figure 2 illustrated a more compact and densely developed mycelium network at pH 6.0 than at the other pH levels. Based on the findings, the culture medium was adjusted to pH 6.0 before sterilization in the subsequent experiments.

#### 3.1.3. Effect of Different Concentrations of Various Nitrogen and Nutrient Sources on *P*. *ostreatus* Mycelial Growth

**Figure 3:**
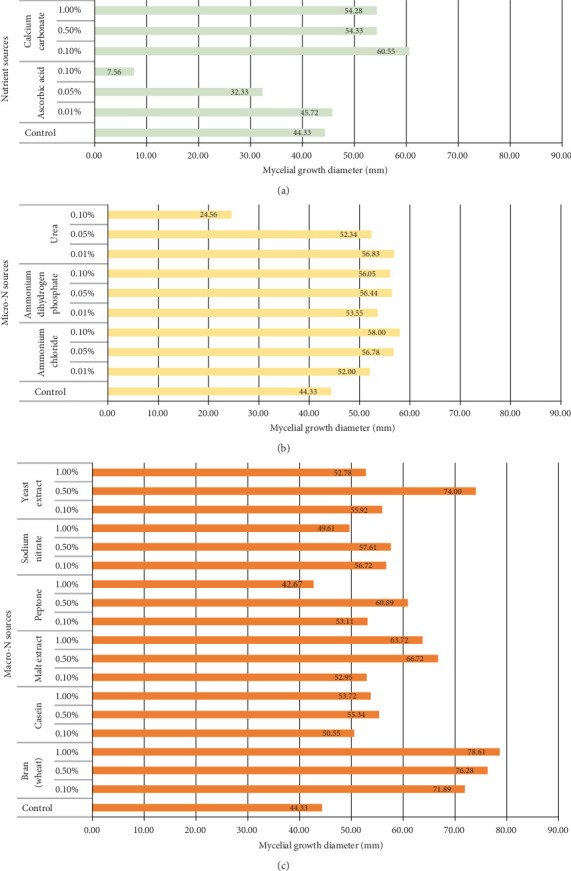
Effect of *P*. *ostreatus* mycelial growth on basal media supplemented with (a) nutrient sources, (b) micronitrogen sources, and (c) macronitrogen sources.

The effects of three inorganic and six organic nitrogen sources, as well as two nutrient sources on the radial growth diameter *P*. *ostreatus* mycelium are presented in Figure 3. On the 6^th^ day of the culture period, the macronitrogen sources (Figure 3(a)): bran-containing (1.00%), yeast-containing (0.50%), malt-containing (0.50%), peptone-containing (0.50%), sodium nitrate (NaNO_3_) (0.50%), and casein-containing (0.50%) medium, facilitated considerable mycelial growth at 78.61, 74.00, 66.72, 60.89, 57.61, and 55.34 mm, respectively. Comparatively, the micronitrogen sources (Figure 3(b)): NH_4_Cl-containing medium (0.10%), urea-containing medium (0.01%), and NH_4_H_2_PO_4_-containing medium (0.05%), facilitated considerable growth at 58.00, 56.83, and 56.44 mm, respectively. The nutrient sources (Figure 3(c)): calcium carbonate (0.10%) and ascorbic acid (0.01%), facilitated considerable growth at 60.55 and 45.72 mm, respectively.

In another study, Hoa and Wang [5] employed *P*. *ostreatus* (strain AG 2041) to study the effect of nitrogen sources and concentrations on mycelium growth. Based on their results obtained on the 6^th^ day of the culture period, the highest mycelium colony diameters were obtained with NH_4_Cl-containing medium at 0.05% (37.00 mm), followed by peptone-containing medium at 1.00% (35.00 mm), and yeast extract–containing medium at 1.00% concentration (34.20 mm), using potato sucrose agar medium. These results may be comparable to our current results since NH_4_Cl, a micronutrient, facilitated the highest growth rate, whereas yeast extract, a macronutrient, facilitated the second-highest growth rate, with the exception of concentrations.

It can be said that different fungal species are impacted differently by nitrogen variations, with too low or too high concentrations inhibiting mycelial growth rate [19]. In the current study, it was determined that both nutrient sources must be supplied at the lowest concentration level to impact the highest mycelial growth. When compared to the control medium, ascorbic-containing medium (micronutrient) at 0.10% concentration significantly inhibited mycelia growth. On the other hand, organic nitrogen sources have been reported to be more effective than inorganic nitrogen in increasing mycelia growth or yield [20], which is consistent with the results of this study, suggesting that *Pleurotus* sp. favors organic nitrogen sources for development.

According to the data illustrated in Figure 3, supplementary nitrogen and nutrient source concentrations that promoted the highest mycelial growth were utilized as optimal sources [5]. These were assessed further in Section 3.2.4 as additional supplements to nutrient-based media for their impact on mycelial protein content.

### 3.2. Liquid-State Culture of *P*. *ostreatus*

#### 3.2.1. The Yield and Structure of Mycelia Cultivated on Various Nutrient-Based Media Sources

Lu et al. [21] stated that the nutrient media source and culture period (days) must be considered when cultivating mycelia. According to the current results in Table 3, it is evident that selecting the precise harvest period of mycelium is essential in attaining the optimal yield. This indicates that the highest mycelial yield not only varies with the media containing different carbohydrate or nutrient sources but also with the cultivation period.

The highest mycelial yield on the 8^th^ day of the culture period was obtained for the Bambara groundnut–based (9.47%) medium and amaranth seed–based (8.90%) medium, for control media. The lowest yield was also obtained on control media, particularly flax seed–based (4.28%) and hemp seed–based (3.21%) media.

On the 10^th^ day of the culture period, the highest mycelial yield was also obtained for the Bambara groundnut–based (15.1%) medium and amaranth seed–based (14.6%) medium, for the control medium. Amaranth seed–based medium was only observed to influence the highest mycelial yield (at 20.1%–21.3%) on the 12^th^ and 14^th^ day of the culture period, followed by Bambara groundnut–based (at 18.5%–20.7%) medium, for control media. In another study, Krupodorova et al. [22] determined that the best culture medium that significantly increased the biomass yield of *P*. *ostreatus* mycelia (HK35) strains on the 14^th^ day of the culture period was obtained by combining amaranth seed flour (waste after CO_2_ extraction) with another media component basis. Based on these findings, it can be stated that amaranth seed flour can increase the mass production of *P*. *ostreatus*.

For the current study, it was determined that in the case of control media, hemp seed–based medium was not efficient in promoting the growth of mycelia, producing the lowest yields (from 3.22% to 8.75%) throughout the culture periods. Similar results were obtained for ragi-based media (both control and glucose-containing media), producing the lowest yields (from 5.73% to 10.7%) consistently for the last three culture periods. The highest yield attributes of amaranth seed–based and Bambara seed–based medium (without glucose) translates into lower production costs and better economic viability [23] of the *P*. *ostreatus* mycelial products in industrial scale.

**Figure 4:**
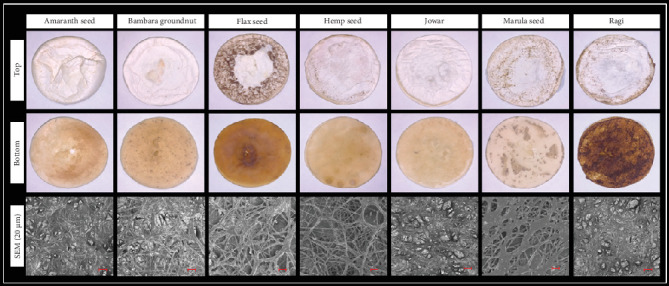
Morphological presentation (top and bottom) of freshly harvested mycelium biomass and scanning electron microscope (SEM) micrograph structure (all scale bars = 20 *μ*m) characterization of lyophilized mycelia from the seven nutrient-based media sources.

Fresh *P*. *ostreatus* mycelium biomass and the structure of a lyophilized filamentous network as impacted by the nutrient-based media source are illustrated in Figure 4. The culture medium composition might influence high-density mycelium networks characterized by finer hyphae variation [4].

It is observed that amaranth seed–based and Bambara groundnut–based medium sources produced comparable compact filamentous networks with fewer open spaces or pores (microscopic openings within the mycelial network). Jowar- and ragi-based medium sources also developed similar structured filamentous networks, which may be due to the similarity of millet types. Marula seed–based medium resulted in mycelia with a thicker filamentous network, with rounded open pores. In contrast, hemp seed–based medium developed mycelia with a relatively thin filamentous network and multiple wide-open pores when compared to other nutrient-based media sources. It has been reported by Sharma et al. [24] that too much porosity can weaken the mycelial network, so an optimal balance is essential.

#### 3.2.2. Effect of the Seven Nutrient-Based Media Sources on *P*. *ostreatus* Mycelial Protein Content

When assessing the impact of mycelial protein concentration, the culture period is fundamentally important (Table 4). On the 8^th^ and 10^th^ days of the culture period, the highest mycelial protein content (approximately ± 39%) was observed for the hemp seed–based medium (control media). Marula seed–based media (control media) produced mycelia with the second highest protein content (ranging from 36.6% to 40.0%) on the 8^th^, 10^th^, and 12^th^ days of the culture period. However, on the 14^th^ day of the culture period, it produced the highest mycelial protein content (42.5%) that was comparable to hemp seed–based media, with no significant difference (*p* > 0.05).

On the 10^th^ and 12^th^ days of the culture period, Bambara groundnut–based (both control and glucose-containing media) and amaranth seed–based media (control) had similar results, producing mycelia with a protein content of approximately ± 29%. On the 14^th^ day of the culture period, Bambara groundnut–based media (both control and media with glucose) produced 28.0% of mycelial protein content, and amaranth seed–based media produced approximately ± 30% of mycelial protein content. The optimum mycelial protein content from these nutrient mediums was observed on the 8^th^ day of the culture period, at 30.3% and 33.4% for Bambara groundnut–based medium and amaranth seed–based media, respectively.

Throughout the cultivation periods, the millet medium types (jowar and ragi based) produced mycelia with the lowest protein content, ranging from 15.6% to 25.3%. This suggested that they are not suitable sources of nutrients for *P*. *ostreatus* mycelium cultivation.

#### 3.2.3. Protein Yield Produced Weights and Variable of Importance in the Projection (VIP) of *P*. *ostreatus* Mycelium

**Figure 5:**
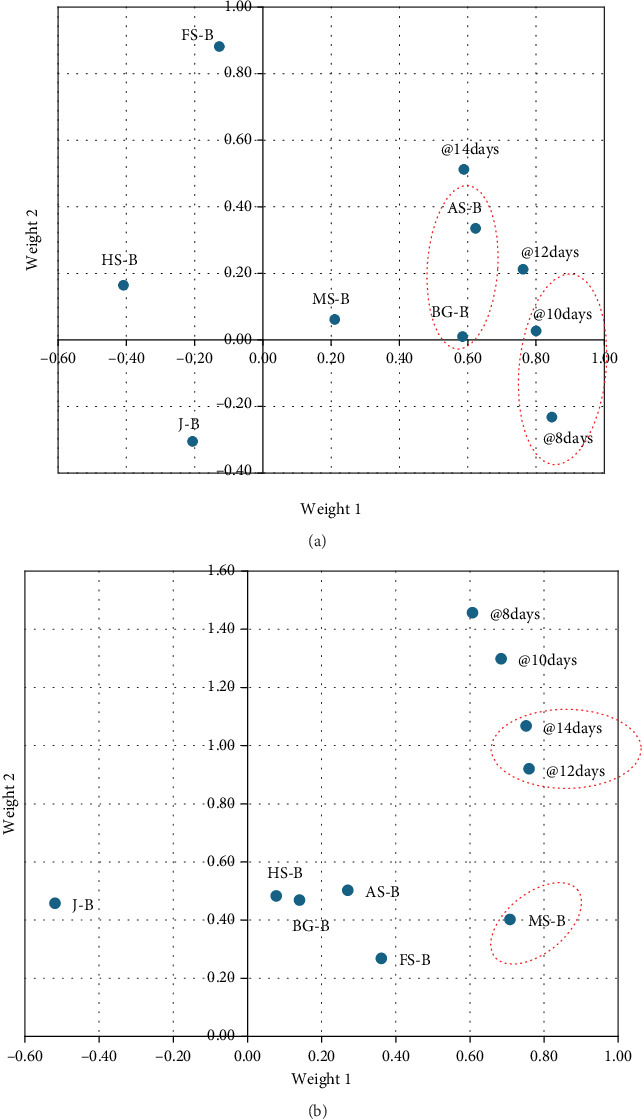
Weight plots for the partial least squares regression (PLS-R) components for mycelial protein yield per hour for (a) control nutrient–based media source and (b) nutrient-based media source with glucose (2%). Variables with the highest weights are in red circles. HS-B: hemp seed–based; J-B: jowar–based; MS-B: marula seed–based; BG-B: Bambara groundnut–based; AS-B: amaranth seed–based mediums.

The results from Sections 3.2.1 and 3.2.2 were used to determine the protein yield over time, which was then used in the generation of a regression model. The individual PLS-R models for each type of nutrient-based media source (control medium and medium with glucose) and culture period (days) are illustrated in Figure 5.

**Figure 6:**
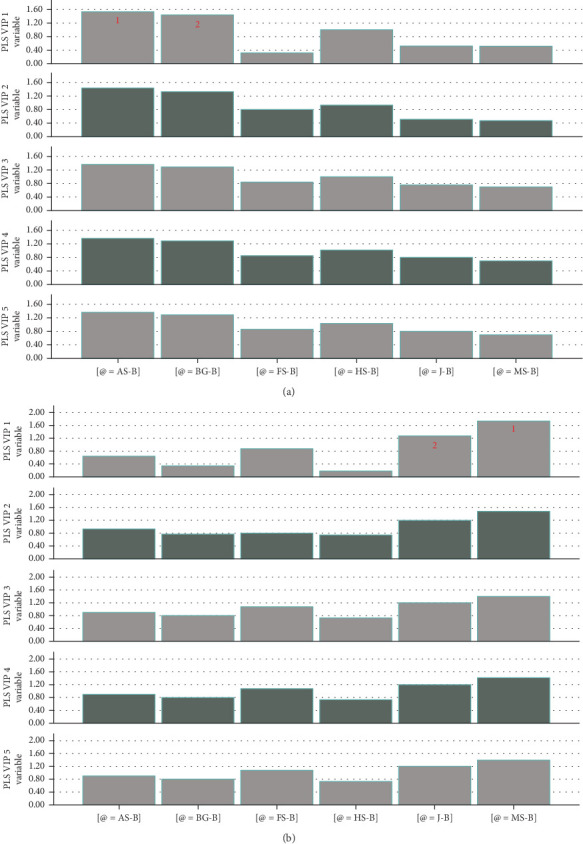
Variable importance for the projection (VIP) of each prediction (a) control nutrient–based media source and (b) nutrient-based media source with glucose (2%). The important predictors with VIPs greater than 1 are consecutively numbered on the bars to show their relative importance. AS-B: amaranth seed–based; BG-B: Bambara groundnut–based; FS-B: flax seed–based; HS-B: hemp seed–based; J-B: jowar–based; MS-B: marula seed–based mediums.

The PLS-R model was also used to predict the VIP for each nutrient-based medium source, in Figure 6. For the control-based medium, amaranth seed–based and Bambara groundnut–based media were of much higher variance on the protein yield, with weights of 0.62 and 0.59, respectively. In the case of flax seed–based, hemp seed–based, and jowar-based mediums, the weights reached −0.13, −0.41, and −0.21, respectively.

The PLS-R model also identified that the 8^th^ day of the culture period was the most inheritable variable, followed by the 10^th^ day, with weights of 0.85 and 0.80, respectively (Figure 5(a)). The VIPs for control media of the nutrient-based media sources were amaranth seed–based (VIP = 1.53) and Bambara groundnut–based medium (VIP = 1.43) (Figure 6(a)).

When modelled for nutrient-based media with 2% glucose, marula seed–based medium with a weight of 0.71 was the nutrient source with the highest variance in protein yield (Figure 5(b)), whereby the 12^th^ and 14^th^ days of the culture period displayed the highest weights of 0.76 and 0.75, respectively (Figure 5(b)). The VIPs in the protein yields of the nutrient-based media were marula seed–based (VIP = 1.74) and jowar-based mediums (VIP = 1.27) (Figure 6(b)). However, the weight for the jowar-based medium reached −0.519 as observed in Figure 5(b).

Table 5 shows the summary of the individual PLS-R models constructed for determining the specific mycelial protein yield for each nutrient-based media source. The goodness of fit (*R*^2^) values for control media were 0.90 and 0.83 for media containing glucose, whereas the cross-validated goodness of prediction (*Q*^2^) values for each variable were also greater than zero, indicating predictive relevance. In reliable models, *R*^2^ values over 0.70 are indicative of a strong relationship between the independent and dependent variables [25]. These results also suggested that the model may accurately predict *P*. *ostreatus* mycelial production when it is cultivated in various sources of nutrient-based media.

In the present study, PLS-R was an efficient method with high prediction scores for evaluating the relative importance of the two major parameters (nutrient-based media source and culture period) on *P*. *ostreatus* mycelial protein yield. The type of nutritional source for cultivating mycelia has a wide range of implications, encompassing economic, environmental, and social aspects. To overcome sustainability challenges related to the scalability, medium sources should be widely available and inexpensive in the local market [4].

Based on the results obtained and described, amaranth seed–based medium and Bambara groundnut–based medium were chosen as the most suitable nutrient-based media sources to cultivate mycelium. Considering that both sources have also been determined to contain sucrose (0.58–0.75 g/100 g) as a primary carbon source [26, 27], which, when employed in medium formulations, reduces dependency on isolated sugars. This, in turn, promotes sustainable research and production approaches. Additionally, it is more efficient to incorporate underutilized crops such as amaranth seeds and Bambara groundnut, which align with sustainability, cultural preservation, and economic diversification principles. They fundamentally promote a more resilient and adaptable agricultural system, which enables to ensure food security [28, 29].

#### 3.2.4. Effect of the Additional Nitrogen and Nutrient Source Supplements on the Protein Content of *P*. *ostreatus* Mycelia

The results from Section 3.2.3 which were used based on the VIPs of mycelia cultivated using control nutrient–based media, were subsequently utilized to improve mycelia production in this section. In addition to the basic growth medium, fungi require a combination of macronutrients and micronutrients, which can be supplemented to enhance the nutritional value of the medium and promote mycelium binding to organic particles [4]. Media composition was supplemented with nine organic (bran, casein, malt extract, peptone, urea, and yeast extract) and inorganic (NaNO_3_, NH_4_Cl, and NH_4_H_2_PO_4_) to optimize the protein content, whereas the two nutrient sources (ascorbic acid and calcium carbonate) were evaluated for their impact on the mycelial protein content. The effect of nitrogen sources was established on the 8^th^ day of mycelial cultivation, the primitive harvestation period (Section 3.2.3).

The results in Table 6 show that the highest mycelial protein content from amaranth seed–based medium was observed in the case of urea-containing medium (38.7%), peptone-containing (37.9%), and NaNO_3_-containing (37.1%) medium, with no significant difference (*p* > 0.05).

The highest mycelial protein content from Bambara groundnut-based medium was observed in the case of yeast-containing (35.9%) and bran-containing (33.6%) medium. For the amaranth seed–based medium, calcium carbonate (CaCO_3_)-containing medium suppressed the mycelial protein content by 0.80%, whereas in the Bambara groundnut–based medium, the protein content was suppressed by 3.80%. While both nutrient sources may impact nutrient availability in the culture medium, they indirectly inhibit the protein expression of the fungus. There is no direct evidence that supports this claim; however, CaCO_3_ was supplemented directly by Mardiana, Astuti, and Abidin [30] as lime to adjust the pH of the substrate and determine its impact on the growth of *P*. *ostreatus* mycelia.

The current study, the assessment of the intricate nutritional composition of each nutritional source and supplementing it with the deficient nitrogen or nutritional source, particularly those that are optimal for *P*. *ostreatus*, has proved that it can significantly improve protein synthesis and overall mycelium development. Of the three supplementary nitrogen sources (urea, peptone, and NaNO_3_) that promoted high protein content in mycelia for amaranth seed–based medium, an assessment of production costs will ensure the economic feasibility of scaled-up cultivation purposes. It is also equally important to consider that in industrial scale production, these should be available as food-grade, animal origin-free (AOF) sources [31].

While NaNO_3_ is valuable in a variety of industrial applications, it poses considerable risks to the environment and water systems, if not managed responsibly [32]. Peptone is known to be derived from animal tissues; however, it can also be obtained from plants or yeast (e.g., phytone peptone, soy peptone 100, and cotton peptone 200) [33]. Both sources have been reported to be expensive, with plant-based peptones having varying compositions and costing more than synthetic ones. Conversely, urea is relatively cost-effective and a readily available source of nitrogen [34]. It was utilized by Shokrkar and Zamani [35] in a fungal culture medium to produce cellulase and high biomass as a protein source.

For these reasons, urea is the preferred nitrogen source in the culture of *P*. *ostreatus* mycelium. Moreover, it is only required at a lower percent concentration (micronutrient) in a culture medium, and it is available as a Food Chemical Codex (FCC) ingredient, making it suitable for all food processes [36]. As a result, mycelia can serve as a sustainable and economical source of alternative protein products.

## 4. Conclusion

The findings of this research revealed several significant physiological features and different nutritional requirements in *P*. *ostreatus* mycelia by using solid surface culture and liquid-state culture methods. These nutritional requirements were valuable in developing the optimal culture medium for consistent and effective enhanced mycelial growth. The culture conditions optimized the production of *P*. *ostreatus* mycelia in 8 days. Using urea as a supplementary organic nitrogen source best optimized the mycelial protein content on an amaranth seed–based medium. These findings represent a significant advancement in the cultivation process used to produce mycelial biomass, which can further be scaled up in industrial processes. This ability to influence *P*. *ostreatus* mycelial biomass as an alternative protein for human consumption may be represented by this information.

## Figures and Tables

**Table 1:** Effect of carbon source and concentration on *P*. *ostreatus* mycelial growth.

**Supplementation**		**Mycelial colony diameter** **(mm/6** days**)**
**Carbon sources**	**Concentration (%)**	
Control	—	23.50 ± 0.44^bc^

Fructose	2	53.57 ± 0.55^h^
	4	54.50 ± 1.13^hi^
	6	45.07 ± 1.01^g^
	8	34.43 ± 0.81^de^

Glucose	2	55.73 ± 1.36^hi^
	4	37.03 ± 1.36^ef^
	6	36.60 ± 1.71^ef^
	8	33.10 ± 1.39^d^

Maltose	2	53.70 ± 1.85^h^
	4	57.33 ± 0.91^i^
	6	53.43 ± 0.81^h^
	8	42.17 ± 0.85^g^

Sorbose	2	38.17 ± 0.15^f^
	4	25.17 ± 1.04^c^
	6	21.37 ± 2.11^b^
	8	16.43 ± 0.78^a^

Sucrose	2	54.83 ± 0.78^hi^
	4	61.27 ± 0.93^j^
	6	61.03 ± 0.40^j^
	8	53.43 ± 0.81^h^

*Note:* Values are means ± SD of triplicate samples. Values within the mycelial colony diameter column followed by the same letters are not significantly different at *p* ≤ 0.05 according to Duncan's multiple range test.

**Table 2:** Effect of the various pH levels on *P*. *ostreatus* mycelial growth over the culture period (days).

**pH level**	**Mycelium colony diameter (mm/day)**	
	**4** ^**th**^ ** day**	**6** ^**th**^ ** day**
pH 4	30.80 ± 0.70^a^	63.89 ± 0.63^b^
pH 5	32.67 ± 0.47^b^	64.33 ± 2.62^b^
pH 6	35.33 ± 0.85^c^	74.61 ± 0.63^c^
pH 7	33.23 ± 0.63^b^	64.35 ± 1.93^b^
pH 8	29.33 ± 0.42^a^	57.56 ± 0.97^a^

*Note:* Values are means ± SD of triplicate samples. Values within the same column of each mycelial growth on each incubation day followed by the same letters are not significantly different at *p* ≤ 0.05 according to Duncan's multiple range test.

**Table 3:** Effect of various nutrient-based media sources on *P*. *ostreatus* mycelial yield over the culture period (days).

**Nutrient media source**	**Yield (%)**			
	**8** ^**th**^ ** day**	**10** ^**th**^ ** day**	**12** ^**th**^ ** day**	**14** ^**th**^ ** day**
*Control*				
Amaranth seed–based	8.90 ± 0.13^de^	14.6 ± 0.38^gh^	20.1 ± 0.25^g^	21.3 ± 1.14^g^
Bambara groundnut–based	9.47 ± 0.41^e^	15.1 ± 1.01^h^	18.5 ± 0.85^fg^	20.7 ± 1.51^fg^
Flax seed–based	4.28 ± 0.26^ab^	7.25 ± 0.75^ab^	10.1 ± 1.88^abc^	16.1 ± 3.81^cde^
Hemp seed–based	3.22 ± 0.26^a^	5.88 ± 0.38^a^	6.88 ± 0.20^a^	8.75 ± 0.43^a^
Jowar–based	7.87 ± 0.13^cde^	10.6 ± 0.89^def^	12.5 ± 0.25^cd^	13.7 ± 0.80^bcd^
Marula seed–based	6.28 ± 1.11^bc^	9.50 ± 0.50^bcd^	11.0 ± 0.25^cd^	12.2 ± 0.38^abc^
Ragi–based	7.27 ± 0.68^cd^	8.25 ± 0.29^abc^	8.38 ± 0.43^bc^	10.7 ± 0.58^ab^
*Glucose (2%)*				
Amaranth seed–based	7.40 ± 1.06^cde^	10.3 ± 0.64^cde^	13.3 ± 0.64^cde^	16.9 ± 1.01^def^
Bambara groundnut–based	7.87 ± 0.41^cde^	12.7 ± 1.61^fg^	13.4 ± 0.92^cde^	16.4 ± 1.00^de^
Flax seed–based	6.27 ± 1.63^bc^	10.0 ± 1.20^cd^	15.4 ± 3.82^def^	18.1 ± 1.70^efg^
Hemp seed–based	6.53 ± 0.23^c^	9.40 ± 0.53^bcd^	10.8 ± 0.69^abc^	13.9 ± 0.81^bcd^
Jowar–based	6.67 ± 0.81^c^	9.33 ± 0.46^bcd^	9.60 ± 1.06^abc^	11.2 ± 0.53^ab^
Marula seed–based	7.53 ± 0.31^cde^	12.5 ± 1.17^efg^	16.7 ± 0.90^efg^	17.3 ± 0.31^defg^
Ragi–based	5.73 ± 0.64^bc^	7.40 ± 0.35^ab^	8.27 ± 0.83^ab^	9.00 ± 0.87^a^

*Note:* Values are means ± SD of triplicate samples. Values within the same column of each mycelial yield followed by the same letters are not significantly different at *p* ≤ 0.05 according to Duncan's multiple range test.

**Table 4:** Effect of the various nutrient-based media sources on *P. ostreatus* mycelial protein content (g/100 g dry weight basis) over the culture period (days).

**Nutrient media source**	**Protein content (%)**			
	**8** ^**th**^ ** day**	**10** ^**th**^ ** day**	**12** ^**th**^ ** day**	**14** ^**th**^ ** day**
*Control*				
Amaranth seed–based	31.4 ± 0.61^e^	29.9 ± 0.75^d^	29.8 ± 0.32^d^	30.7 ± 0.20^f^
Bambara groundnut–based	30.3 ± 0.40^e^	29.2 ± 0.47^d^	29.6 ± 0.57^d^	28.0 ± 0.72^e^
Flax seed–based	31.5 ± 0.06^e^	36.2 ± 0.53^gh^	36.4 ± 1.45^f^	35.8 ± 0.17^h^
Hemp seed–based	39.7 ± 0.30^h^	39.4 ± 1.11^i^	42.3 ± 0.47^g^	42.3 ± 0.36^j^
Jowar–based	25.3 ± 0.31^d^	24.6 ± 0.35^c^	24.8 ± 1.00^c^	25.2 ± 0.25^d^
Marula seed–based	36.6 ± 0.21^g^	37.5 ± 0.31^h^	40.0 ± 0.57^g^	42.5 ± 0.20^j^
Ragi–based	18.3 ± 0.23^b^	19.2 ± 1.37^b^	18.6 ± 0.15^b^	18.5 ± 0.10^b^
*Glucose (2%)*				
Amaranth seed–based	33.4 ± 0.15^f^	32.1 ± 0.62^e^	32.2 ± 0.30^e^	29.5 ± 0.45^f^
Bambara groundnut–based	25.4 ± 0.15^d^	28.9 ± 0.10^d^	29.6 ± 1.21^d^	28.0 ± 0.15^e^
Flax seed–based	31.2 ± 0.83^e^	34.1 ± 0.46^f^	34.7 ± 0.46^f^	33.5 ± 0.15^g^
Hemp seed–based	33.6 ± 0.26^f^	33.3 ± 0.10^ef^	34.7 ± 1.07^f^	34.2 ± 0.10^g^
Jowar–based	22.2 ± 0.78^c^	24.3 ± 0.59^c^	25.3 ± 1.16^c^	23.3 ± 1.06^c^
Marula seed–based	34.0 ± 0.59^f^	34.8 ± 0.30^fg^	35.0 ± 0.75^g^	37.7 ± 0.47^i^
Ragi–based	15.6 ± 0.10^a^	15.6 ± 0.10^a^	15.9 ± 0.45^a^	15.2 ± 0.06^a^

*Note:* Values are means ± SD of triplicate samples. Values within the same column of each mycelial protein content followed by the same letters are not significantly different at *p* ≤ 0.05 according to Duncan's multiple range test.

**Table 5:** Summary of partial least regression (PLS-R) model for mycelial protein yield. The *R*^2^ (goodness-of-fit) and *Q*^2^ (cross-validated-goodness-of-prediction) over the PLS-R components.

**Variable**	**R** ^2^	**Q** ^2^	**Component**	**Explained variance in ** **Y**	**Cumulative explained variance in ** **Y**
				**%**	
Control media	0.90	0.86	1	62.3	62.3
			2	8.70	71.0
			3	23.7	94.2
			4	0.40	94.6
			5	0.20	94.8

Media with glucose	0.83	0.87	1	48.8	48.8
			2	33.1	81.9
			3	7.90	89.8
			4	1.30	91.1
			5	0.10	91.2

**Table 6:** Protein content (g/100 g dry weight) of *P*. *ostreatus* mycelium harvested from two nutrient-based media sources supplemented by additional nitrogen and nutrient sources.

**Supplementation**	**Mycelial protein content (%)**	
	**Amaranth seed–based**	**Bambara groundnut–based**
*Nitrogen sources*		
Bran (wheat)	34.5 ± 1.59^b^	33.6 ± 1.08^de^
Casein	34.2 ± 0.56^b^	30.7 ± 0.78^bc^
Malt extract	34.6 ± 0.36^b^	31.6 ± 1.35^cd^
Peptone	37.9 ± 0.64^c^	32.8 ± 1.10^cd^
NaNO_3_	37.1 ± 0.29^c^	28.3 ± 1.37^ab^
NH_4_Cl	33.7 ± 0.75^b^	28.7 ± 0.81^ab^
NH_4_H_2_PO_4_	33.7 ± 0.84^b^	32.0 ± 0.32^cd^
Urea	38.7 ± 0.55^c^	31.7 ± 0.80^cd^
Yeast extract	33.8 ± 0.32^b^	35.9 ± 0.25^e^
*Nutrient sources*		
Ascorbic acid	30.6 ± 0.60^a^	31.7 ± 0.97^cd^
CaCO_3_	29.9 ± 1.00^a^	26.5 ± 0.38^a^

*Note:* Values are means ± SD of triplicate samples. Values within the same column of each mycelial yield followed by the same letters are not significantly different at *p* ≤ 0.05 according to Duncan's multiple range test.

Abbreviations: CaCO_3_, calcium carbonate; NaNO_3_, sodium nitrate; NH_4_Cl, ammonium chloride; NH_4_H_2_PO_4_, ammonium dihydrogen phosphate.

## Data Availability

The data used to support the findings of this study are included within the article. However, any other information required is available from the corresponding author upon request.

## Supporting Information

Supporting Information Additional supporting information can be found online in the Supporting Information section. Additional supporting information can be found online in the Supporting Information section. Table S1: Various nitrogen and nutrient source measurements for supplementation on nutrient-based media.
