# Supplementary material for: Effect of Culture Media on the Yield and Protein Content of Pleurotus ostreatus (Jacq.) Kumm Mycelia
Source: Int J Food Sci. 2024 Dec 27;2024:5562732. doi: 10.1155/ijfo/5562732 (PMC11698607; doi:10.1155/ijfo/5562732)
Supplement: Supporting Information — Additional supporting information can be found online in the Supporting Information section. Additional supporting information can be found online in the Supporting Information section. Table S1: Various nitrogen and nutrient source measurements for supplementation on nutrient-based media. [file 5562732.f1.docx]

**SUPPLEMENTARY TABLE 1** Various nitrogen and nutrient source measurements for supplementation on nutrient-based media

| **Supplements** | **Concentration (%)** |
| --- | --- |
| **Macro-nitrogen sources** | |
| Bran (wheat) | 1.00 |
| Casein | 0.50 |
| Malt extract | 0.50 |
| Peptone | 0.50 |
| Sodium nitrate | 0.50 |
| Yeast extract | 0.50 |
| **Micro-nitrogen sources** | |
| Ammonium chloride | 0.10 |
| Ammonium dihydrogen phosphate | 0.10 |
| Urea | 0.01 |
| **Nutrient sources** |  |
| Ascorbic acid | 0.01 |
| Calcium carbonate | 0.10 |
